# Supplementary material for: Rieske iron-sulfur protein induces FKBP12.6/RyR2 complex remodeling and subsequent pulmonary hypertension through NF-κB/cyclin D1 pathway
Source: Nat Commun. 2020 Jul 15;11:3527. doi: 10.1038/s41467-020-17314-1 (PMC7363799; doi:10.1038/s41467-020-17314-1)
Supplement: Supplementary file 1 — Supplementary Information [file 41467_2020_17314_MOESM1_ESM.docx]

**Rieske iron-sulfur protein induces FKBP12.6/RyR2 complex remodeling and subsequent pulmonary hypertension through NF-κB/cyclin D1 pathway**

**Authors:** Lin Mei, Yun-Min Zheng, Tengyao Song, Vishal R. Yadav, Leroy Joseph, Lillian Truong, Sharath Kandhi, Margarida M. Barroso, Hiroshi Takeshima, Marc A. Judson and Yong-Xiao Wang

**Supplement Information:**

**1. Supplementary Data**

- Supplementary Figure 1

- Supplementary Figure 2

- Supplementary Figure 3

- Supplementary Figure 4

- Supplementary Figure 5

- Supplementary Figure 6

- Supplementary Table 1

- Supplementary Table 2

**Supplementary Figure 1.**

**
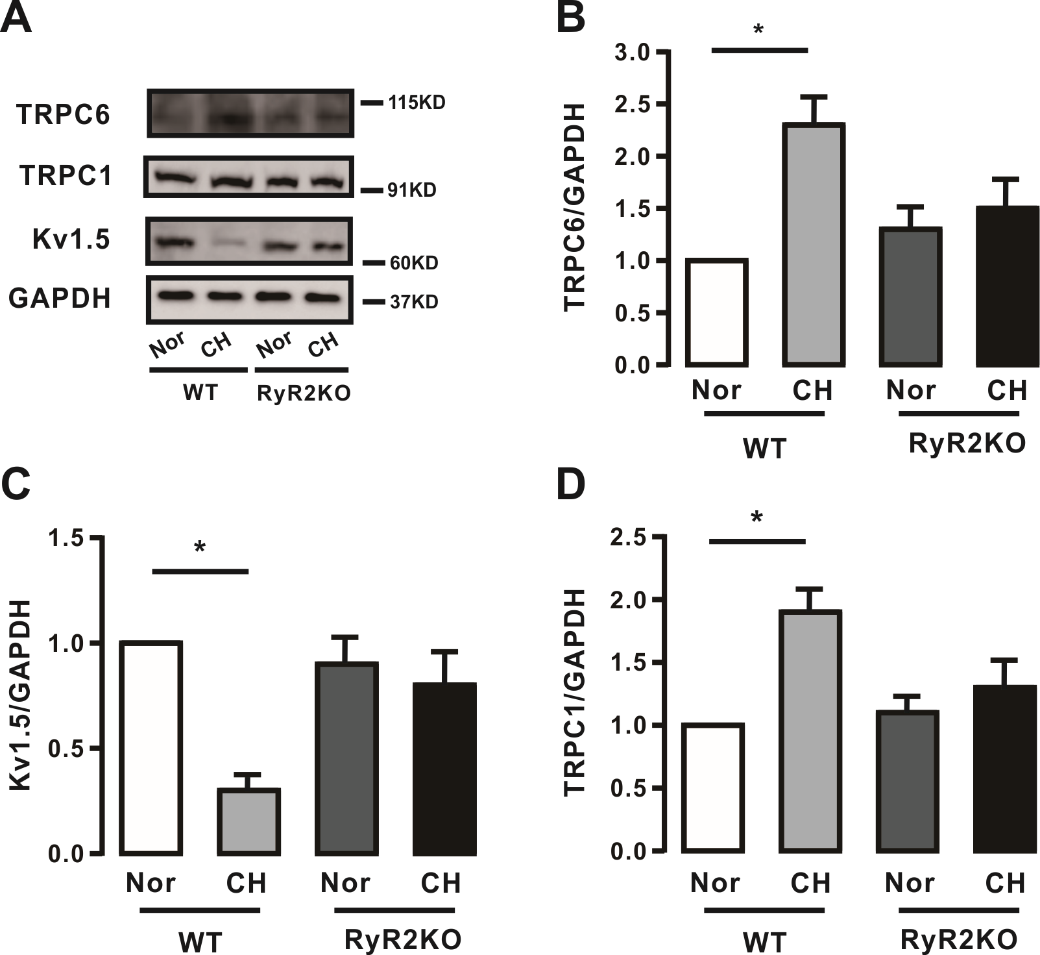
**

**RyR2 KO inhibits ion-channel expression in PASMC. A)** Representative western blot shows the up-regulation of TRPC1,6 and down-regulation of K_v_1.5 have been restored in RyR2 KO mice. **B-D)** Bar graphs describe the summarized results of TRPC1,6 and Kv1.5 expression in PH model (n=3 independent studies, 5 mice per group). Data are expressed as mean ± standard error. (*P<0.05, using one-way ANOVA test)

**Supplementary Figure 2.**

**
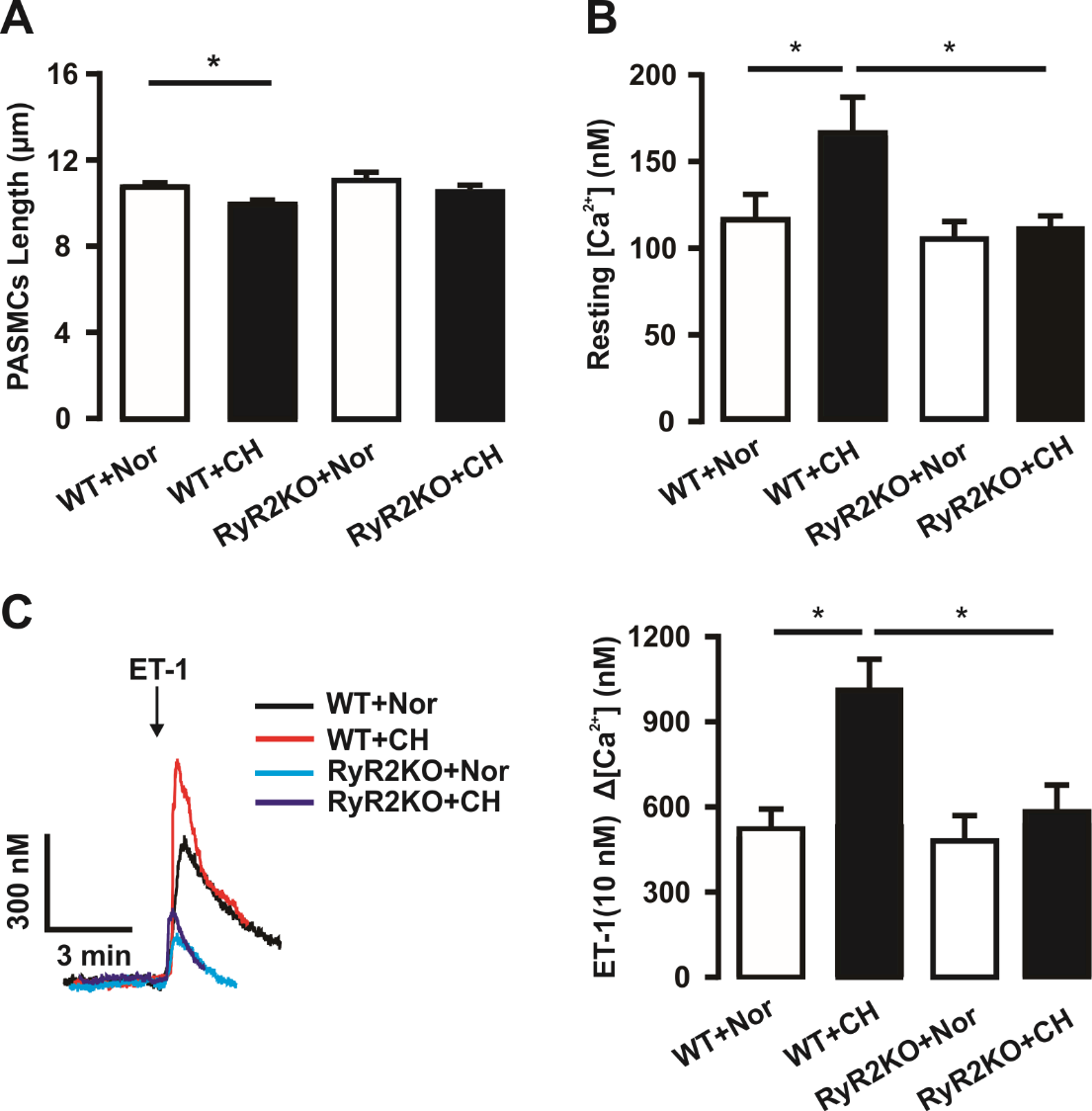
**

**RyR2KO PAMSC is resistant to CH-induced dysfunction of Ca^2+^ signaling. A)** 3 weeks CH results in a significant contraction (shortening) of PASMC. Bar graph depicts the mean length of PASMC reflecting the contractile status (n=7 independent studies, 100 cells per study). **B)** Summary of elevated resting [Ca^2+^]_i_ level after exposure to 3 weeks CH. PASMC are loaded with fura-2/AM (5µM) (n=7 independent studies, 100 cells per study). **C)** Original recording of calcium measurement under stimulation of ET-1. Application of maximal dosage of ET-1 (10nM) cannot induce comparable [Ca^2+^]_i_ peak in RyR2 KO model after CH (n=6 independent studies, 70 cells per study). Data are expressed as mean ± standard error. (*P<0.05, using one-way ANOVA test)

**Supplementary Figure 3.**

**
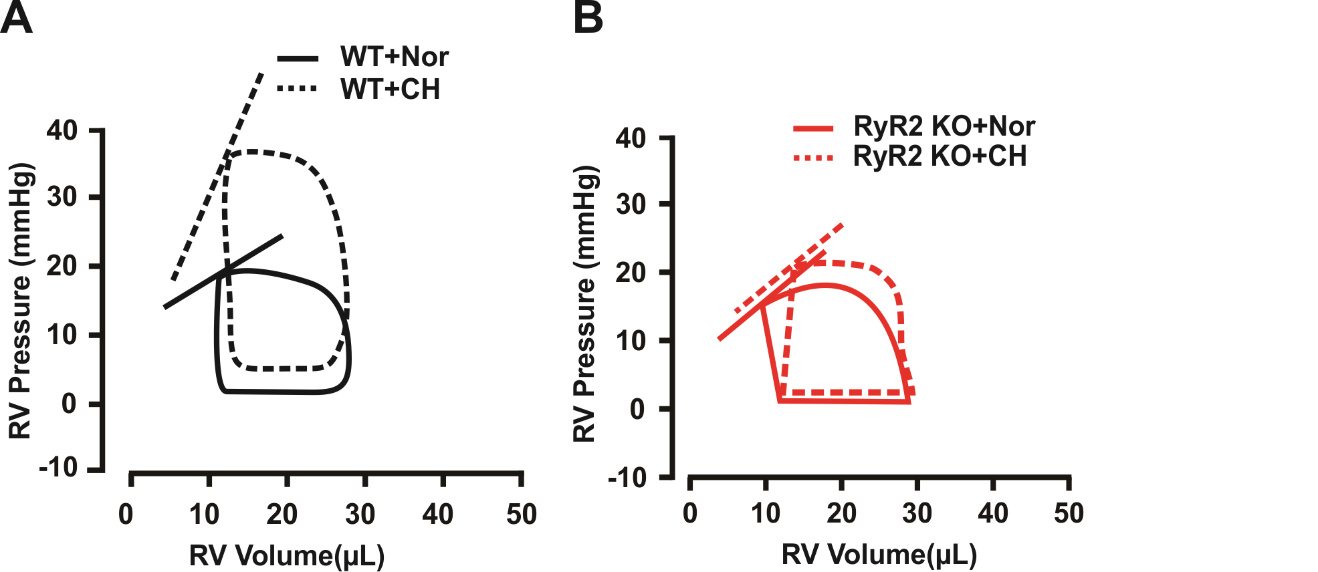
**

**Representative pressure-volume (P-V) loops.** **A)** P-V loops are measured using 1.2F conductance pressure-volume catheter (Scisense, Canada) in wildtype (WT) mice exposed to chronic hypoxia (CH). Data are obtained during alteration of preload by occlusion of inferior vena cava. **B)** Representative P-V loops in RyR2KO mice exposed to CH.

**Supplementary Figure 4.**

**
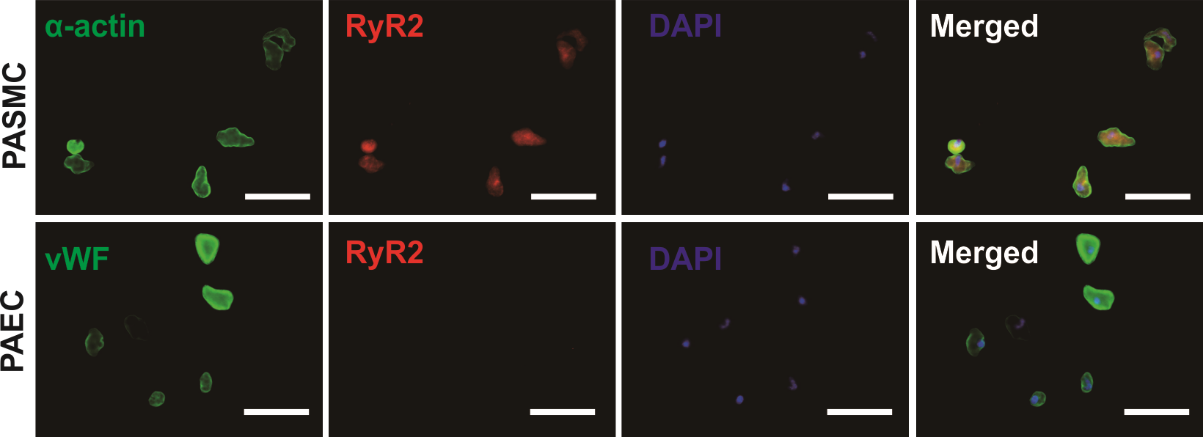
**

**Representative immunofluorescence staining**. Smooth muscle immunofluorescence marker (α-actin) and endothelial cell marker (von Willebrand factor, vWF) show RyR2 is mainly expressed in PASMC rather than pulmonary arterial endothelial cell (PAEC). Bar scale: 20μm.

**Supplementary Figure 5.**

**
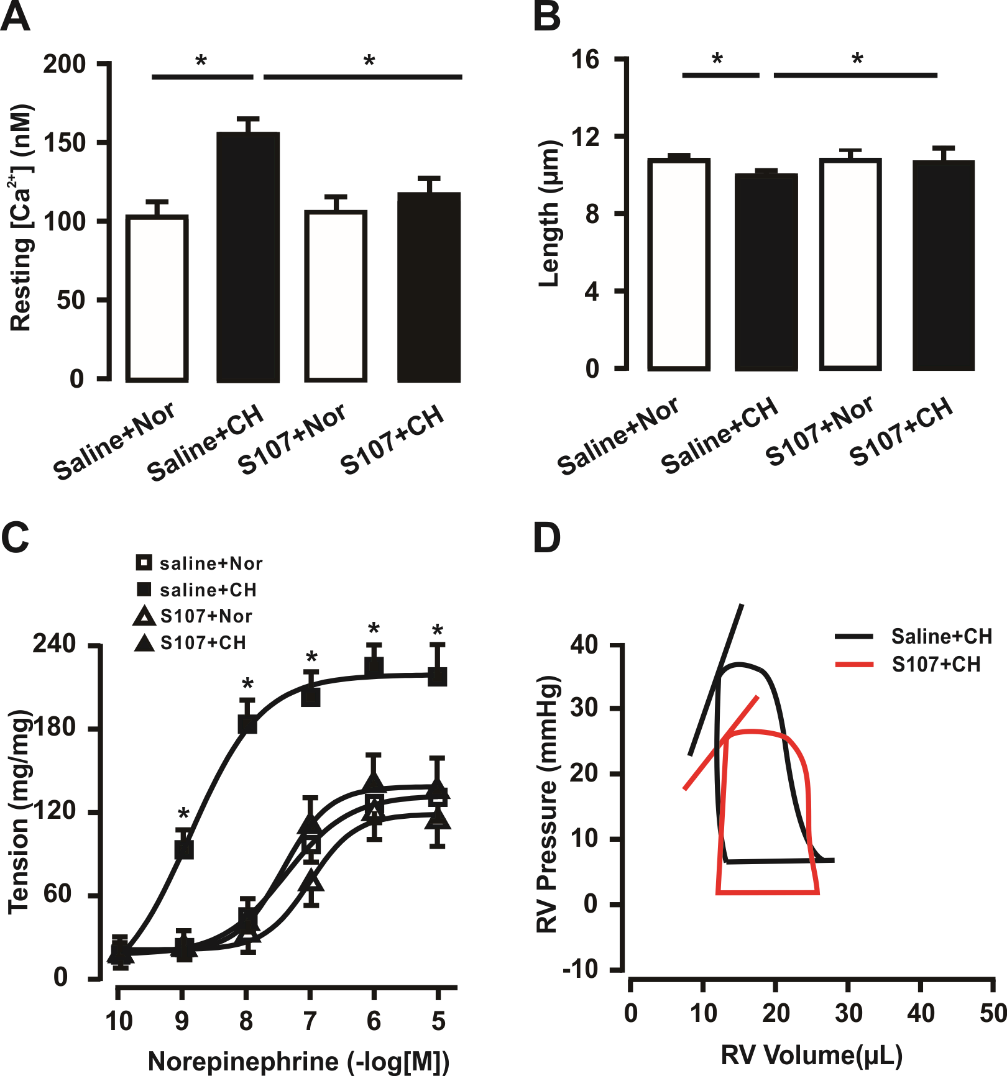
**

**S107 CH-induced PH and associated alteration of Ca^2+^ signaling. A)** Summary of resting [Ca^2+^]_i_ levels in S107 treating models after exposure to CH (n=7 independent studies, 80 cells per study). **B)** Effect of S107 on the contractility of PAs (n=7 independent studies, 80 cells per study). **C)** Concentration-response curves for norepinephrine-induced muscle contraction are obtained under normoxic (Nor) and CH PA strips from control (saline) and S107 treatment mice (n=7 independent studies, 5 PA strips per study). **D)** Representative pressure-volume loops and ESPVR in the group of S107 after chronic hypoxia exposure. Data are obtained during alteration of preload by occlusion of inferior vena cava. Data are expressed as mean ± standard error. (*P<0.05, using one-way ANOVA test)

**Supplementary Figure 6.**

**
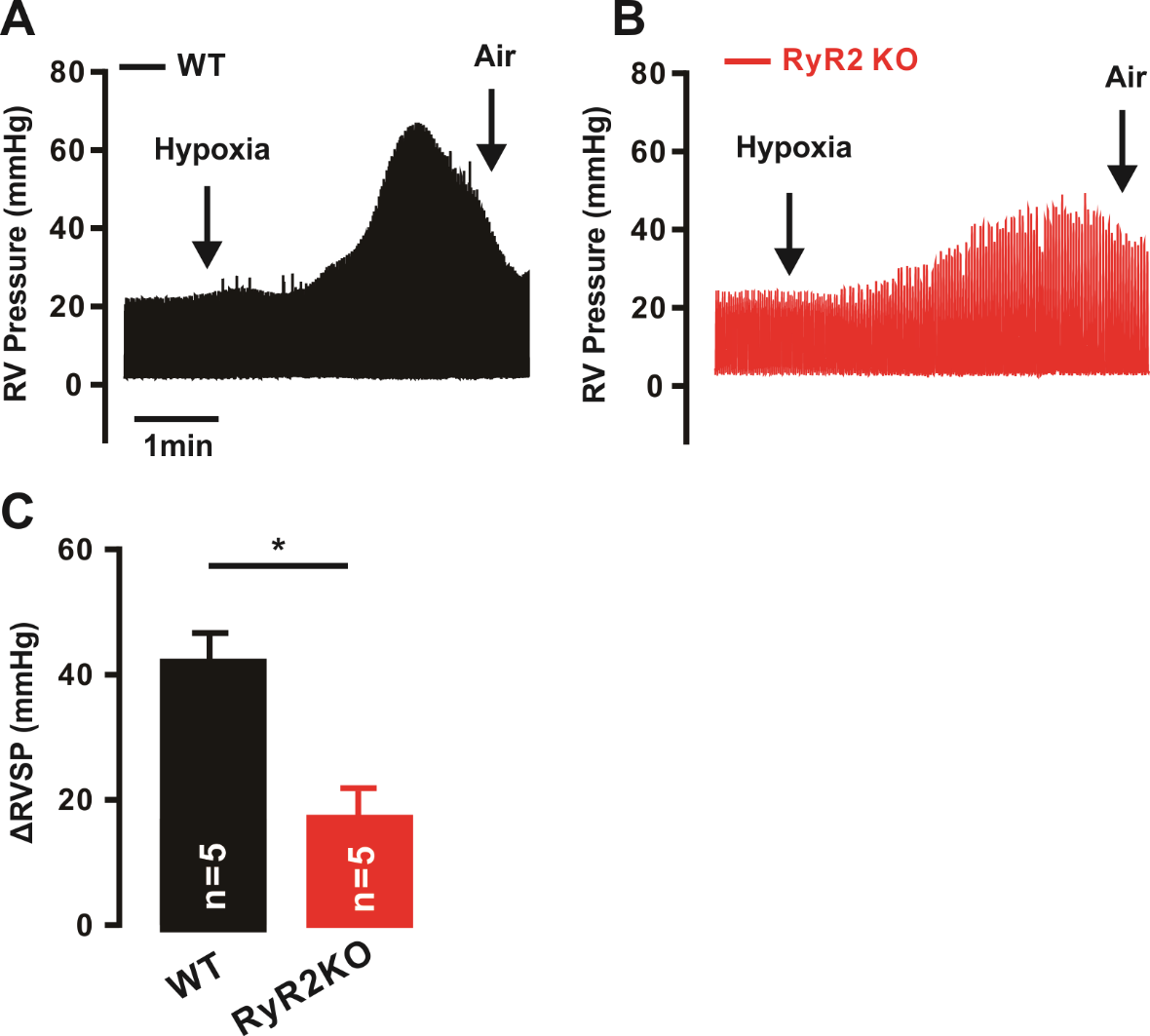
**

**Effect of acute hypoxia on RyR2 KO mice. A-B)** Original traces of in vivo pulmonary arterial constriction after exposure to acute hypoxia (1% O_2_) measured by 1.2F pressure catheter. **C)** Analyzed data of ∆RVSP from RyR2 KO mice after exposure to acute hypoxia (n=4 independent studies, 5 mice per study). Data are expressed as mean ± standard error. (*P<0.05, using Student’s *t* test)

**Supplementary Table 1. Hemodynamic parameters of systolic and diastolic function derived from right ventricular pressure-volume relationship**

| **Parameter** | **WT+Nor (n=8)** | **WT+CH**  **(n=9)** | **RyR2KO+**  **Nor(n=6)** | **RyR2 KO+**  **CH(n=8)** | |
| --- | --- | --- | --- | --- | --- |
| **HR(beats/min)** | 545±35 | 521±30 | 495±25 | 507±30 |  |
| **RVSP(mmHg)** | 19.5±2 | 35.5±2.5* | 18.5±1 | 21.5±1.5 |  |
| **RVDP(mmHg)** | 1.5±0.5 | 3.5±1.0* | 0.8±0.3 | 1.5±0.6 |  |
| **EDV(μl)** | 27.0±3.0 | 29.2±2.0 | 27.0±3.0 | 25.7±3.3 |  |
| **SW(mmHg·μl)** | 250±50 | 410±70* | 230±40 | 280±55 |  |
| **SV(μl)** | 14.3±1.7 | 11.2±1.5* | 13.2±2.1 | 13.6±1.9 |  |
| **CO(ml/min)** | 7.9±2.1 | 5.8±1.5* | 6.6±2.0 | 6.9±2.2 |  |
| **EF(%)** | 54±7 | 38±5* | 51±5 | 52±7 |  |
| **dP/dt(mmHg/s)** | 2010±900 | 3350±740* | 2350±520 | 2400±764 |  |
| **-dP/dt(mmHg/s)** | -1800±410 | -1700±350 | -2050±450 | -1550±460 |  |
| **Ees(mmHg/μl)** | 1.3±0.4 | 2.1±0.5* | 1.1±0.4 | 1.5±0.4 |  |
| **Ea(mmHg/μl)** | 1.3±0.5 | 4.2±0.7* | 1.0±0.4 | 1.6±0.3 |  |
| **Ees/Ea** | 1.0±0.1 | 0.5±0.08* | 1.1±0.1 | 0.9±0.3 |  |

dP/dt_max_ and dP/dt_min_, maximum and minimum derivative of pressure; Ees, ventricular end-systolic elastance; Ea, effective arterial elastance. Data are expressed as mean ± standard error. *P<0.05 compared with control group, using one-way ANOVA test.

**Supplementary Table 2. Hemodynamic parameters of systolic and diastolic function derived from right ventricular pressure-volume relationship**

| **Parameter** | **Saline+Nor (n=8)** | **Saline+CH**  **(n=9)** | **S107+**  **Nor(n=7)** | **S107+**  **CH(n=7)** | |
| --- | --- | --- | --- | --- | --- |
| **HR(beats/min)** | 523±32 | 540±25 | 563±29 | 520±30 |  |
| **RVSP(mmHg)** | 20.3±2 | 36.0±2.5* | 21.0±1.5 | 24.1±1.5 |  |
| **RVDP(mmHg)** | 0.75±0.2 | 3.0±0.9* | 1.0±0.3 | 1.5±0.6 |  |
| **EDV(μl)** | 25.0±3.0 | 27.5±2.5 | 25.7±2.6 | 26.5±3.5 |  |
| **SW(mmHg·μl)** | 290±56 | 376±60* | 287±44 | 297±55 |  |
| **SV(μl)** | 15.0±2.3 | 10.4±2.0* | 14.4±2.5 | 13.7±2.0 |  |
| **CO(ml/min)** | 7.9±2.0 | 5.6±1.8* | 8.2±2.4 | 7.2±2.3 |  |
| **EF(%)** | 61±8 | 39±5* | 57±6 | 52±8 |  |
| **dP/dt(mmHg/s)** | 1938±600 | 3340±790* | 2075±500 | 2495±660 |  |
| **-dP/dt(mmHg/s)** | -1647±520 | -2063±530 | -2208±490 | -2408±480 |  |
| **Ees(mmHg/μl)** | 1.0±0.3 | 2.1±0.4* | 1.4±0.4 | 1.5±0.5 |  |
| **Ea(mmHg/μl)** | 1.2±0.4 | 5.4±0.8* | 1.4±0.5 | 1.6±0.3 |  |
| **Ees/Ea** | 0.9±0.1 | 0.4±0.1* | 1.1±0.2 | 1.0±0.3 |  |

dP/dt_max_ and dP/dt_min_, maximum and minimum derivative of pressure; Ees, ventricular end-systolic elastance; Ea, effective arterial elastance. Data are expressed as mean ± standard error. *P<0.05 compared with control group, using one-way ANOVA test.
