# Supplementary material for: Rieske iron-sulfur protein induces FKBP12.6/RyR2 complex remodeling and subsequent pulmonary hypertension through NF-κB/cyclin D1 pathway
Source: Nat Commun. 2020 Jul 15;11:3527. doi: 10.1038/s41467-020-17314-1 (PMC7363799; doi:10.1038/s41467-020-17314-1)
Supplement: Supplementary file 3 — Reporting Summary [file 41467_2020_17314_MOESM3_ESM.pdf]

nature research

Corresponding author(s): Yong-xiao Wang (NCOMMS-19-17078)
Last updated by author(s): May 20, 2020

Reporting Summary

Nature Research wishes to improve the reproducibility of the work that we publish. This form provides structure for consistency and transparency in reporting. For further information on Nature Research policies, see Authors & Referees and the Editorial Policy Checklist .

Statistics

For all statistical analyses, confirm that the following items are present in the figure legend, table legend, main text, or Methods section.

n/a	Confirmed	
☐	☒	The exact sample size (*n*) for each experimental group/condition, given as a discrete number and unit of measurement
☐	☒	A statement on whether measurements were taken from distinct samples or whether the same sample was measured repeatedly
☐	☒	The statistical test(s) used AND whether they are one- or two-sided<br>*Only common tests should be described solely by name; describe more complex techniques in the Methods section.*
☐	☒	A description of all covariates tested
☐	☒	A description of any assumptions or corrections, such as tests of normality and adjustment for multiple comparisons
☐	☒	A full description of the statistical parameters including central tendency (e.g. means) or other basic estimates (e.g. regression coefficient) AND variation (e.g. standard deviation) or associated estimates of uncertainty (e.g. confidence intervals)
☐	☒	For null hypothesis testing, the test statistic (e.g. *F*, *t*, *r*) with confidence intervals, effect sizes, degrees of freedom and *P* value noted<br>*Give P values as exact values whenever suitable.*
☒	☐	For Bayesian analysis, information on the choice of priors and Markov chain Monte Carlo settings
☒	☐	For hierarchical and complex designs, identification of the appropriate level for tests and full reporting of outcomes
☒	☐	Estimates of effect sizes (e.g. Cohen's *d*, Pearson's *r*), indicating how they were calculated

Our web collection on statistics for biologists contains articles on many of the points above.

Software and code

Policy information about availability of computer code

Data collection	Methods, softwares and equipment for data collection are provided in methods section.
Data analysis	Origin 8.5 was used for graph generation and data analyses.

For manuscripts utilizing custom algorithms or software that are central to the research but not yet described in published literature, software must be made available to editors/reviewers. We strongly encourage code deposition in a community repository (e.g. GitHub). See the Nature Research guidelines for submitting code & software for further information.

Data

Policy information about availability of data

All manuscripts must include a data availability statement. This statement should provide the following information, where applicable:
- Accession codes, unique identifiers, or web links for publicly available datasets
- A list of figures that have associated raw data
- A description of any restrictions on data availability

The data supporting the findings of this study are available from the corresponding author upon request.

Field-specific reporting

Please select the one below that is the best fit for your research. If you are not sure, read the appropriate sections before making your selection.

☒ Life sciences ☐ Behavioural & social sciences ☐ Ecological, evolutionary & environmental sciences

For a reference copy of the document with all sections, see nature.com/documents/nr-reporting-summary-flat.pdf

October 2018

Life sciences study design

All studies must disclose on these points even when the disclosure is negative.

Sample size	Sample size were determined based on the experimental setting and availability of the specimen. For animal models, these are largely depend on the relevant experience in our lab and previous studies by others. Due to the limitation of the human tissue samples, we only studied the samples that were available. All of our findings were only reported to be conclusive and successful if statistical significance was obtained. All numbers and type of replicates are mentioned in the figure legend and can be found in the source data file.
Data exclusions	Data points were only excluded in few circumstance if they are identified as outliers due to technical failures during experimental process.
Replication	All our findings were repeated at least 3 times in different biological replicates, as well as technical independently measurements.
Randomization	No randomization method was used.
Blinding	For the quantification of IHC staining and pathological evaluation, the investigator were blinded to ensure an unbiased interpretation. Other studies were not performed in a blinded fashion due to the risk of confusion in handling.

Reporting for specific materials, systems and methods

We require information from authors about some types of materials, experimental systems and methods used in many studies. Here, indicate whether each material, system or method listed is relevant to your study. If you are not sure if a list item applies to your research, read the appropriate section before selecting a response.

Materials & experimental systems

n/a	Involved in the study	
☐	☒	Antibodies
☐	☒	Eukaryotic cell lines
☒	☐	Palaeontology
☐	☒	Animals and other organisms
☐	☒	Human research participants
☐	☒	Clinical data

Methods

n/a	Involved in the study	
☒	☐	ChIP-seq
☒	☐	Flow cytometry
☒	☐	MRI-based neuroimaging

Antibodies

Antibodies used

FKBP12.6 antibody (PA1-026A), ABR Affinity Bio-Reagents
Rieske (RISP) antibody (PA5-21420), Thermo Fisher Scientific
anti-RyR2 antibody (MA3-916), ABR Affinity Bio-Reagents
p50 antibody (E-10) sc-8414, Santa Cruz Biotech
cyclin D1 antibody (A-12), sc-8396, Santa Cruz Biotech
IκB-α antibody (B-3), sc-373893, Santa Cruz Biotech
α-actin antibody (1A4), sc-32251, Santa Cruz Biotech
vWF antibody (H-300), sc-14014 , Santa Cruz Biotech
GAPDH antibody (0411) sc-47724, Santa Cruz Biotech
p65 antibody (#3034), Cell signaling Technology, Inc
Lamin A antibody (ab2559), Abcam
anti-Ki67 antibody (ab15580), Abcam
Kv1.5 antibody (APC-150), Alomone Labs,
TRPC1 antibody (ACC-118), Alomone Labs
TRPC6 antibody (ACC-017), Alomone Labs

Validation

Validations are based on the datasheets from the manufacturers.

Eukaryotic cell lines

Policy information about cell lines

Cell line source(s)	Primary cells are obtained from each mouse model.<br>293FT cell line for viral transfection was bought from Thermo Fisher Scientific Ltd.
Authentication	None of the cell lines used were authenticated.

Mycoplasma contamination	All cells were tested negative for mycoplasma.
Commonly misidentified lines (See ICLAC register)	*Name any commonly misidentified cell lines used in the study and provide a rationale for their use.*

Animals and other organisms

Policy information about studies involving animals; ARRIVE guidelines recommended for reporting animal research

Laboratory animals	The studies used laboratory mouse strain C57BL/6, FKBP12.6 knockout mice and RyR2 smooth-muscle specific knockout mice. Male mice with 12-16 weeks old were used for experiments.
Wild animals	The study did not involve wild animals.
Field-collected samples	The study did not involve samples collected from the field.
Ethics oversight	All animals procedures were approved by the Institutional Animal Care and Use Committee of Albany Medical College. The study protocol number is 17-07003.

Note that full information on the approval of the study protocol must also be provided in the manuscript.

Human research participants

Policy information about studies involving human research participants

Population characteristics	Human pulmonary aterial specimens were obtained from the National Disease Research Interchange (NDIR) or Albany Medical Center Hospital and used according to the protocols approved by the Institutional Review Board for the Protection of Human Subjects in Research at Albany Medical College. Pulmonary hypertension was diagnosed by physical exam, medical history review and various tests. The pulmonary arterial specimens from age-matched subject who did not have any detectable cardiovascular diseases were used control.
Recruitment	N/A
Ethics oversight	The donor tissue donation was performed by NDIR or Albany Medical Center Hospital in accordance with national law, Good Clinical Practice/International Conference on Harmonisation guidelines and local ethics committee.

Note that full information on the approval of the study protocol must also be provided in the manuscript.

Clinical data

Policy information about clinical studies

All manuscripts should comply with the ICMJEguidelines for publication of clinical research and a completedCONSORT checklist must be included with all submissions.

Clinical trial registration	N/A
Study protocol	N/A
Data collection	N/A
Outcomes	N/A
